# When east meets west: a qualitative study of barriers and facilitators to evidence-based practice in Hunan China

**DOI:** 10.1186/s12912-018-0295-x

**Published:** 2018-06-20

**Authors:** Wendy Gifford, Qing Zhang, Shaolin Chen, Barbara Davies, Rihua Xie, Shi-Wu Wen, Gillian Harvey

**Affiliations:** 1 School of Nursing, Faculty of Health Sciences, University of Ottawa, 451 Smyth Road, Ottawa, ON K1H 8M5 Canada; 2 Nursing Best Practice Research Center, 451 Smyth Road, Ottawa, ON K1H 8M5 Canada; 3 School of Nursing, Hunan University of Medicine, 492 Jinxinan Road, Huaihua, Hunan China; 4 Nanhai Hospital, Southern Medical University, 45 ZhenXing Road, Lishui Town, Nanhai District, Foshan, 528244 Guangdong China; 5 OMNI Research Group, Department of Obstetrics, Gynecology and Newborn Care, Faculty of Medicine University of Ottawa, Ottawa, Canada; 6 Clinical Epidemiology Program, Ottawa Hospital Research Institute, Ottawa, Canada; 7 Department of Epidemiology and Community Medicine, University of Ottawa, 501 Smyth Box 51, Ottawa, ON K1H 8L6 Canada; 8 Adelaide Nursing School, The University of Adelaide, Adelaide, Australia; 9 Alliance Manchester Business School, University of Manchester, Manchester, UK

**Keywords:** Qualitative study, Evidence-based practice, Barriers, Facilitators, China

## Abstract

**Background:**

Research into evidence-based practice has been extensively explored in nursing and there is strong recognition that the organizational context influences implementation. A range of barriers has been identified; however, the research has predominantly taken place in Western cultures, and there is little information about factors that influence evidence-based practice in China. The purpose of this study was to explore barriers and facilitators to evidence-based practice in Hunan province, a less developed region in China.

**Methods:**

A descriptive qualitative methodology was employed. Semi-structured interviews were conducted with staff nurses, head nurses and directors (*n* = 13). Interviews were translated into English and verified for accuracy by two bilingual researchers. Both Chinese and English data were simultaneously analyzed for themes related to factors related to the evidence to be implemented (Innovation), nurses’ attitudes and beliefs (Potential Adopters), and the organizational setting (Practice Environment).

**Results:**

Barriers included lack of available evidence in Chinese, nurses’ lack of understanding of what evidence-based practice means, and fear that patients will be angry about receiving care that is perceived as non-traditional. Nurses believed evidence-based practice was to be used when clinical problems arose, and not as a routine way to practice. Facilitators included leadership support and the pervasiveness of web based social network services such as Baidu (百度) for easy access to information.

**Conclusion:**

While several parallels to previous research were found, our study adds to the knowledge base about factors related to evidence-based practice in different contextual settings. Findings are important for international comparisons to develop strategies for nurses to provide evidence-based care.

**Electronic supplementary material:**

The online version of this article (10.1186/s12912-018-0295-x) contains supplementary material, which is available to authorized users.

## Background

As the largest group of healthcare professionals, nurses play an essential role in delivering evidence-based practice (EBP) for positive patient outcomes [1]. However, gaps exist between recommendations for EBP and the actual care nurses provide in practice [2, 3]. A range of barriers has been identified, however, and while factors that influence EBP have been extensively explored by researchers in many countries, this has predominantly taken place in Western cultures; little information exists about EBP in China [4, 5]. As a relatively new concept, a recent scoping review (*n* = 95 articles) found barriers not commonly documented in western countries [6]. For example, the values embedded in traditional Chinese culture continue to have a strong influence on the ways in which Chinese people think and behave, which in turn affects the ways healthcare is delivered. Reverence for authority and obedience to superiors typically influences Chinese nurses’ motivation to change their practice independently, particularly as they are perceived to have little decision-making autonomy and low professional status [5, 7]. Order and harmony are expected social behaviours in China, and many people expect healthcare to be rooted in traditional Chinese practices that may not have a strong evidence base [8, 9]. Such reverence for authority and expectation for tradition impedes nurses from implementing EBP or considering it a routine way of practice [8, 9].

Large health and healthcare inequities exist between China’s more developed urban provinces that are largely coastal and generate a large proportion of the country’s wealth [10], and its less developed rural provinces that lack resources and infrastructure to meet healthcare needs [11, 12]. Across different provinces, inconsistencies also exist in nurses’ understanding and acceptance of EBP [13]. Research has indicated that nurses in some provinces of China are more advanced in understanding, accepting and implementing EBP when compared to other provinces where healthcare systems are less developed [13].

Effecting change across healthcare settings and organizations can be challenging. With recognition that the contextual factors influence EBP, there has been recent interest in factors that influence the successful implementation of EBP in mainland China. The majority of research has been quantitative in design and few studies have explored nurses’ experiences of the EBP implementation process [6]. Strategies for implementing EBP have a higher chance of success if they are tailored to known contextual barriers and facilitators both internal and external to the practice settings [14].

The Ottawa Model of Research Use (OMRU) is an implementation framework based on planned change theories that outline the interrelationships and complex organizational dimensions of translating research or new knowledge into practice [15]. The OMRU describes factors that influence the process of implementing research evidence into practice, classifying barriers and facilitators at the levels of the: 1) innovation or evidence to be implemented, 2) potential adopters or healthcare practitioners who will use the evidence and 3) practice environment in which the evidence will be used [15]. However, limited knowledge exists about nurses’ perceptions of factors that hinder or facilitate EBP in China, particularly in less developed regions where healthcare is less developed and resources are scarce.

Qualitatively understanding Chinese nurses’ perceptions of factors that influence the implementation processes of EBP is important to develop strategies for organizational leaders and decision-makers to improve the effectiveness of patient care for positive patient outcomes. To this end, we undertook a qualitative study to understand nurses’ perceptions of the factors that influence nurses’ implementation of EBP in Hunan province, a less developed region in China [16]. The following research questions were addressed:

1. What sources of the knowledge do nurses use to make decisions in their clinical practice?
2. What are the barriers and supports for clinical nurses to use research evidence in their daily practice?

## Methods

Drawing on principles of naturalistic inquiry, a qualitative descriptive study was conducted [17]. Descriptive qualitative research is valuable to explore the experiences of a phenomenon when a comprehensive summary is desired [17]. The aim of qualitative descriptive studies is a descriptive summary of the information in the data desired. Descriptive qualitative analysis is considered the method of choice when straight descriptions of phenomena are desired [17].

### Participants

Convenience sampling was first initiated at a two day “China-Canada” colloquium on best practices in nursing education and practice held in Hunan province where academic researchers in nursing, epidemiology and medicine from Canada and China were featured. Over 400 nurses and healthcare leaders from Hunan province, a mountainous province in south central China, attended. Hunan is a largely agricultural with approximately 60% of the 68.2 million residents living rural lives [18]. Confucianism values are prominent, with the virtues of loyalty, etiquette, ritual and customs strongly influencing the behaviours and social structures of Hunan society [19].

Information about the study was distributed, and interested participants identified themselves to the bilingual research assistants who were present. Snowball sampling was used to further recruit participants after the colloquium ended.

### Data collection

Data were collected through individual, face-to-face semi-structured interviews between November and December 2015 in Hunan province. All interviews were conducted in Mandarin by two bilingual researchers. The principal investigator (WG) did not speak Mandarin but was present for the first five interviews with a bilingual researcher who translated participant responses as the interview progressed, allowing for probes and clarification of meanings and context.

Interviews were conducted with staff nurses, head nurses and senior nursing directors. The interview guide consisted of open-ended questions regarding participants’ views about the type of knowledge they used to make practice decisions, and their experiences of using research evidence to inform practice. Examples of interview questions included: What are the main sources of knowledge that you use to make clinical decisions? From your experience, what would you say are the barriers or challenges for nurses to use research evidence in their clinical decisions? What are some of the facilitators or supports? The full interview schedule can be found in Additional file 1.

Interviews averaged between 40 and 60 min, were audio-recorded, transcribed verbatim into Chinese and translated into English by two bilingual researchers from Hunan province (QZ, SC). The same two researchers independently crosschecked the translation and came to consensus on contextual meanings. Different bilingual investigators (XR, SWW) provided confirmation of the translation and back-translation; any discrepancies were discussed with the research study team until consensus was reached. Both Chinese and English transcriptions were imported together into QSR NVIVO© 10 qualitative software.

### Ethical considerations

Ethical approval was obtained from University of Ottawa Research Ethics Board, and recognized by the Hunan University of Medicine in China. Ethical principles of informed consent, anonymity and right to withdraw were maintained throughout the study. Written information about the study was reviewed with each participant by a Chinese research assistant and verbal consent to participate was received from all participants prior to recording the interviews.

### Data analysis

Analysis proceeded as a group process with the principal investigator (WG) and the two bilingual researchers from China (QZ, SC), hereafter referred to as the ‘analysis team.’ Transcripts were reviewed numerous times to ensure familiarization with the data. The analysis team reviewed the English and Chinese data together and discussed the explicit and implicit meanings in response to the interview questions. If agreement could not be reached about meaning, a fourth party was consulted until consensus was reached.

Qualitative content analysis was informed by procedures described by Graneheim and Lundman [20]. Data were initially coded by the analysis team into meaning units and codes using relevant words and phrases of participants in both English and Chinese. Next, English codes for each of the research questions were collapsed into inductive sub-categories. Barriers and facilitators to EBP were then classified into three broad deductive categories based on the OMRU: 1) Innovations, 2) Potential Adopters and 3) Practice Environment [15].

The analysis process was iterative, with ongoing and simultaneous examination of the Chinese and English data and codes to ensure the analysis team captured appropriate meanings. Larger meetings with the research team were held throughout the analysis to confirm interpretations and findings.

## Results

### Description of participants

Thirteen nurses from tertiary and community hospitals participated in the study (see Table 1). All participants were female with more than five years’ work experience.

**Table 1:** Participant positions and previous awareness of EBP

	Community hospital (*n* = 6)	Tertiary hospital (*n* = 7)	Total (*n* = 13)
Position			
Director	1	1	2
Head Nurse	2	4	6
Staff nurse	3	2	5
Previous awareness of EBP	2 (33%)	4 (57%)	6 (46%)

### Sources of knowledge

To inform their clinical practice decisions-making, nurses described using multiple sources of knowledge that included colleagues (peers, head nurses and doctors), educational seminars and conferences, original nursing education, social media applications (apps) and the internet web service “Baidu.” WeChat, a mobile text and voice messaging service developed in China, was consistently detailed as a primary source for accessing information to make practice decisions, as described by a head nurse in a community hospital:

*Baidu Search or website of Nursing Association, and the public channel “nurses learning notes” in WeChat, where there are some new views about nursing practice or research. (Source 9 Community)*

Participant believed the information they found through their social media apps and on Baidu was accurate, trustworthy and reliable.

### Awareness of EBP

Less than half of the sample (*n* = 6/13; 46%) was aware of the concept of EBP, and only two of the six participants from community had heard of it. Directors and head nurses were more aware than clinical staff.

*We have not started to implement evidence-based nursing practice yet. I heard the concept of EBP a few years ago when I worked in [a tertiary hospital]. I know that the concept has been around for many years, however, implementing EBP has not been long, even in tertiary hospitals. I know little about EBP and there are less complex nursing problems in community that need it. (Source 7 Community)*

For participants who had heard of EBP, it was not perceived to be a routine way of providing care. Rather, EBP was believed to be a way of practice for addressing individualized clinical problems when they could not be dealt with through established routines and traditional ways. This perception did not vary between community and tertiary settings. Respondents believed medical practice was more research-based than nursing, and that tertiary hospitals provided more evidence-based care than community hospitals.

*Nursing is less important than medical science in China. Our hospital leaders do not support us to do research… We just want to do the things required by the superior leaders, and make sure no medical errors happen. (Source 6 Urban)*

### Barriers to EBP

Barriers to applying research evidence in everyday practice were expressed as they related to the innovation, potential adopters and practice settings. A summary of barriers are presented in Table 2.

**Table 2:** Barriers to evidence-based practice

Innovation	
• Lack of evidence written in Chinese language	
• Not enough details in guidelines	
Potential Adopters	
Nurse-related	
• Fear of patients’ and families’ reacting to something new or non-traditional	
• Lack of awareness, knowledge and skills	
• Negative attitudes and beliefs toward EBP	
Patient-related	
• Lack of money	
• Lack of trust	
Practice Environment	
• Lack of leadership support	
• Little/no opportunities for EBP education and training	
• Limited resources (physical and human)	

### Innovation

Respondents reported that the majority of nursing research journals and guidelines were published in English, and therefore a barrier to EBP was the lack of readable and understandable research evidence in Chinese. When guidelines were available, some felt they did not have enough details about how to apply it in practice.

### Potential adopters

#### Nurse-related barriers

Many respondents reported fearing patients and families if they did something that was considered new or non-traditional to what was expected, such as a new practice based on research evidence. Nurses described fear of being blamed or assaulted both physically and verbally if patients had a bad outcome, and this fear discouraged nurses from considering doing something that could be perceived as different to or outside the traditional practices. All nurses interviewed described some level of fear of patients and families, as one participant related:

*We tend to be conservative here; we will not use some new medicine or new nursing practices until the practices are generally accepted by everyone, because if there is a bad outcome, the patient will be angry and blame us. (Source 9 Community)*

Participants spoke about a lack of knowledge and skills of clinical nurses about finding, accessing and understanding research evidence, exemplified as follows:

*Nurses’ scientific research knowledge base is very low; therefore, they do not know how to find or appraise evidence or use. (Source 4 Community)*

*But I guess the ordinary clinical nurses may not know what to do, just know the assignments which head-nurse asked them to do. The head-nurses told them if there are some problems, they should use EBP approach to solve them. They feel it is difficult to understand and accept. The reason for this phenomenon may be nurses’ training has not been done well. The Nursing Department of the hospital carried out some training lectures, but most of nurses who attended were head-nurses, most ordinary nurses were busy in clinical care; they could not attend.* (Source 1 Urban)

Holding negative attitudes and beliefs towards research in general was another barrier described by participants. Most respondents reported that they and their nursing colleagues preferred to practice in ways they had learnt in nursing school or that were consistent with routines in their practice settings. EBP was described as being difficult to understand, and participants did not believable that the evidence was always applicable in China:

*… it is much easier to use the traditional method. … The [research] evidence may work for some patients, but may not work for patients in China. (Source 2 Urban)*

#### Patient-related barriers

Participants identified costs to patients and their inability to afford the best care as a barrier to EBP:

*Patients and their families worry about the expense. For example, according to the evidence, we may use a better material to help patients’ recovery, but they cannot afford it. (Source 3 Urban)*

Nurses spoke about patients’ lack of trust when doing things that were considered different or unfamiliar to traditional care. This was emphasized when caring for the elderly patients, where communication difficulties were present:

*Most of our patients are elderly, so it’s hard to communicate with them…. Sometimes, because of generational culture gap, old patients do not trust us, and they do not want to see a change in routine based on something new. (Source 10 Community)*

### Practice setting

Lack of leadership support from head nurses and hospital administrators was a barrier to EBP and included an absence of leaders with EBP knowledge to promote, encourage and facilitate EBP:

*Another main barrier is the way of management. If there was someone to take the responsibility of EBP and educating nurses for EBP, implementation would be much better in clinical practice. (Source 4 Community)*

*Even some nursing leaders do not know what EBP means or how to appraise and implement the evidence. If EBP is needed in clinical practice, nurses will have to learn it by themselves. (Source 7 Community)*

Participants also expressed senior leaders’ lack of understanding and commitment to the implementation of EBP:

*...if the senior leaders of the hospital do not stress on the issues [EBP], why do the clinical nurses do it? Some senior leaders of the hospital do not pay attention to this issue. Evidence-based practice has not been fully carried out at present… some of them [nursing managers] do not even understand the inner meaning of evidence-based practice, they just say it. They know it is priority, but they don’t know how to do it in practice. (Source 3 Urban)*

Participants indicated there were little or no opportunities for EBP education and training, and limited resources for nurses to implement new evidence-based practices, as emphasized:

*Our hospital’s condition is not good enough, and the equipment are not advanced, so evidence-based practice cannot be implemented. (Source 10 Community)*

Limited resources included an insufficient number of nursing staff, resulting in heavy workloads. Participants felt that they had limited time during their scheduled working hours to provide patient care and EBP was seen as extra work:

*Chinese nursing conditions are different from other countries. Chinese nurses’ basic workload is very heavy; we do not have enough time to practice based on new evidence. (Source 4 Community)*

### Facilitators of EBP

Facilitators to EBP in nursing were seldom discussed, however, respondents identified two facilitators related to the potential adopters (i.e. nurses), and four related to the practice setting (Table 3).

**Table 3:** Facilitators to evidence-based practice

Potential Adopter	
• Understanding that EBP improves patient care • Belief that EBP improves credibility of nursing	
Practice Setting	
• Education and training	
• Leadership promotion and support of EBP	
• Presence of EBP team	
• Mechanism to access evidence	

### Potential adopter

Participants emphasized that EBP would increase the credibility of the nursing profession, and bring recognition to the importance of nursing practice for positive patients outcomes.

*I believe evidence-based practice is a very effective way to improve nursing… I believe EBP working method will avoid many medical disputes or contradictions with medical staff…and the evidences will direct our nursing work in future. For example, a patient suffers from pressure ulcer, if we find out some evidences and accumulate these evidences…when we come across another similar patient, we can find out the best nursing method quickly. If we can work this way, I believe it is helpful for our clinical nursing. (Source 4 Community)*

*Now, we head-nurses believe it [EBP] is a wonderful scientific method. If we encounter some problems in clinical, we will search literatures and find evidence, apply these evidence to address problems, then evaluate outcomes or effects. I feel this kind of work method is perfect for nursing (Source 3 Urban)*

### Practice setting

Participants described nursing leadership at many levels as an important EBP facilitator. The role of the government was emphasized in both urban and community hospitals to set the stage for conducting and using research in practice:

*The Ministry of Health can develop some rules about doing research in community hospitals, so the leaders of our hospital will support using research; from the patient's point of view, it is beneficial to them, and nurses will get pleasure and fulfillment from this and increase our professional self-identity. Everybody likes a good work environment, so that we can work in a good mood and work more efficiently. (Source 9 Community)*

Leadership to develop international collaborations has assisted with raising awareness of the importance of EBP and the resources required to provide quality nursing care:

*The collaborations, which are not just among personnel but increasingly with nursing associates and governments with other countries, improves the Chinese government to change their decisions so they know the importance of giving more human resources and more support for the nurses - hire more nurses - so the nurses can do the right job and feel good and strong. Secondly, now our country is advocating for medical reform and high-quality nursing care programs. (Source 3 Urban)*

Senior nursing leaders played a significant role in bringing awareness to organizational leaders regarding the importance of EBP in nursing:

*Nursing care is paid more attention to than before in our country now, and so does our hospital leadership. The nursing group plays a great role in influencing the decisions of hospital leadership… to improve the awareness of EBP in nursing… and there is someone to lead us to improve. …the facilitators mainly include two aspects: hospital leadership’s attention and improvement of nurse education level. (Source 2 Urban)*

*Hospital leadership is important as well; they should pay attention to the hospital future planning and medical safety management. (Source 10 Community)*

The presence of clinical nursing leaders was discussed as a necessary direction to increase awareness and engage staff in research activities that included conducting and using research in practice, as discussed by a head nurse in a large urban hospital.

*The government set up a “Clinical Nursing Key Specialty Program,” which is similar with doctors,’ and they give some funding. Our hospital gained about 3 million RMB from this program for supporting nursing research, training nurses, sending nurses to go abroad for studying and communicating with Taiwan and Hong Kong… The aim of the research is to direct the practice, implement into practice. (Source 3 Urban)*

Education was further described as necessary to facilitate EBP in China. In addition, social media and electronic platforms, which are integral to people’s daily lives, were consistently detailed as a primary resources for accessing information to make practice decisions, as described by a head nurse in a community hospital:

*Baidu Search or website of Nursing Association, and the public channel “nurses learning notes” in WeChat (a mobile text and voice messaging communication service developed by Tencent in China), where there are some new views about nursing practice or research. (Source 9 Community)*

## Discussion

In this study, nurses in a less-developed province in mainland China discussed the knowledge they used to make practice decisions, and the barriers and facilitators to EBP. While several parallels to previous research were found, our study revealed new and important findings that have not been previously reported. Our findings are discussed as they relate to the innovation, the individual and the organization.

### The innovation

One unique finding was the use of mobile social media apps by nurses and other healthcare professionals as a source of knowledge to inform patient care. Fast-paced technological developments that link mobile apps to smartphones and tablet computers are integral to everyday life in China [21]. Previous research has shown that medical professionals’ use of mobile technology was beneficial to making evidence-based decisions and lowering error rates [22, 23]. However, despite all participants in this study regularly using electronic mobile technology at work, they infrequently used it to inform practice decisions, nor did they question the accuracy or reliability of the information they found on popular social media sites.

In 2016, a 21-year-old college student in China died after receiving an experimental cancer treatment that was promoted on a hospital website through the Chinese search engine Baidu [24]. The student’s death drew widespread international attention when the family accused Baidu of promoting false medical information that was supported by staff at the hospital. The ubiquitous use of electronic mobile technology underscores the need for nurses to have critical appraisal knowledge and skills to safely use information from electronic sources to inform their practice decisions. This is particularly relevant today where information from electronic technology is increasingly part of people’s everyday lives. Incorporating evidence-based decision-making tools from trustworthy sources into electronic mobile devices is a promising strategy for increasing EBP nursing in China.

### The individual

Our study found that, in general, bedside nurses had a limited awareness and understanding of EBP and how it could improve care and outcomes. Using research evidence to routinely inform practice decisions was not considered standard practice as traditional ways were deemed the most desirable, and many nursing leaders considered EBP as a way to manage problems when traditional practice failed. This finding provides further insights into previous survey research that showed Chinese nurses do not use research findings in their daily practice decisions [25, 26] and lacked understanding of EBP [27].

To use research evidence routinely in practice, nurses must first be aware of EBP and then have the knowledge and skills to access, appraise, and integrate evidence with patient preferences and clinical experience [27]. Results of our study showed that participants lacked awareness and knowledge of EBP, particularly in community settings. While nurses from tertiary hospitals had greater awareness of EBP, they lacked the knowledge of how to find, appraise and judicially apply the evidence. With nursing research poorly developed in China [28–30], many nurses lack knowledge of the research process and do not understand how research could improve nursing care and outcomes [30–32].

The need to increase awareness and knowledge of EBP in China is an important first step to attenuate barriers and enable evidence-based nursing. This is consistent with an updated systematic review (*n* = 45 articles) that indicated that beliefs and attitudes, information seeking and education influenced nurses’ use of research [33]. In our study, respondents had negative beliefs and attitudes about EBP, and preferred to practice the way they had learned or according to the expected tradition. These findings are similar to cross-sectional survey studies conducted in other non-western countries. Farokhzadian et al. [34] found that nurses in Iran (*n* = 182) had unfavorable attitudes towards EBP, and Lai, Teng, & Lee [35] had similar findings in Malaysia. In our study, nurses stated they did not believe that research evidence was applicable to their settings because the research was not conducted in China, and they would not adopt EBP as routine practice unless they could see positive results on patient care and outcomes.

EBP was seen as a useful way to increase the credibility of the nursing profession and recognize nursing’s contributions to quality patient care. Using research evidence to inform practice was emphasized as a way to recognize nursing as a science-based profession, improving nursing’s credibility, particularly with physicians. These results concur with findings from studies in Iran [36] and Germany [37] that highlight EBP as a way of strengthening understanding of nurses’ contributions to patient care. Aiken et al. [38] showed nursing care was significantly associated with patient mortality and length of stay in nine European countries, emphasizing the role nurses have in influencing patient care and outcomes.

Lack of research studies and evidence-based guidelines in Chinese was identified as a barrier to EBP, and this finding has been similarly reported in other non-English-speaking countries [27, 39–41]. With English being the international language of science [42], the majority of nursing research is published in English; however, less than 1 % of people in China are able to fluently understand it [43]. Translating evidence-based guidelines into Chinese could help overcome language obstacles to accessing research evidence in China [4, 44]. In Canada, the Registered Nurses’ Association of Ontario (RNAO) has produced evidence-based guidelines for nursing care that are freely available on their website (http://rnao.ca/bpg/language), and a number are being translated into Chinese. Interestingly, participants in our study felt that guidelines did not have enough details related to the evidence, which is inconsistent with a survey of almost 1500 nurses in Hong Kong that found guidelines and research reports had too much information [45].

A unique finding in our study was the fear of patients and families that nurses had when doing something new or different from what is expected. This fear is arguably accentuated by the perceived lack of organizational support nurses received when patients and families were unsatisfied with their care or outcomes [5]. Nurses in China face increasingly heavy workloads and stressful work environments that affect the quality of patient care [46]. The lack of leadership support they perceived in our study may partially explain why they are hesitant to implement new or non-traditional practices.

Our findings provide contextual data on work environment barriers within the Chinese healthcare context that may contribute to nurses’ use of evidence in practice.

Patient involvement is an integral part of EBP; however, participants described difficulties getting patients involved in care because of factors such as cultural traditions with aging patients. Reports indicate that the Chinese medical system is not fully prepared to manage the aging trend in China and fails to meet healthcare demands due to lack of resources and expertise [12]. This includes the costs patients assume because of lack of socially funded healthcare, particularly in rural areas where inequalities exist in healthcare services [47].

In our study, the cost of care was identified as problematic for patients; health reforms are providing wider coverage of costs to alleviate financial burdens caused by illness [48, 49], and government policies have been developed for rural farmers to have access to basic health services [12]. However, lax governance regulatory and enforcement systems are an obstacle to reducing out-of-pocket spending for healthcare, as healthcare delivery often concentrates on services that generate the most profit rather than the evidence base [50]. The influence of government health reforms to facilitate EBP and improve health outcomes for people in China warrants further investigation.

### The organizational setting

Using research evidence for decision-making in practice is a complex process and factors related to the organizational setting consistently explain a large amount of the variance in EBP [5]. Lack of leadership support, heavy workload, no opportunities for continuing education and lack of specialized staff were organizational factors found to be barriers in this study.

Previous studies indicate that leadership support is related to successful implementation of EBP [2, 51–53]. In our study, staff nurses reported the need for support from head nurses and, in turn, head nurses needed support from their superiors, which were often hospital directors. Leadership and the management of healthcare in China have been built on traditional hierarchical structures in which hospital administrators and medical doctors have higher status and authority over nurses [5].

Confucian principles of loyalty, obedience and respect for the social order, where everyone knows the behaviours expected in relation to others, have strongly influenced the norms and behaviours of Chinese people [54, 55]. Full support from superiors like head nurses, directors and physicians is required for nurses to deviate from the status quo and make changes to routine practices, such as EBP. In light of the hierarchies embedded in Chinese cultural values, nurses lack the authority to initiate change; therefore, leadership support is essential to facilitate EBP in China [5]. As healthcare reform continues in China [56], leadership is required to promote EBP as a routine part of nursing practice. Leadership commitment and support are positively related to the implementation of EBP in western countries [2, 51, 57].

The leadership of bedside nurses, as champions for EBP, has also been shown to facilitate implementation [58]. Establishing key personnel to promote and share research findings with staff can support nurses’ understanding and implementing of EBP. An organizational intervention that included having designated champions to facilitate staff accessing and understanding of research evidence influenced nurses to use research findings in their practice [59].

Prior studies have indicated that a lack of education is a barrier to EBP [34, 60]. Most participants in our study stated that they had not had any professional education about EBP, and felt that educational opportunities would enhance their inquiry and appraisal skills to practice EBP. With little focus on research in nursing school, the majority of nurses in China have not received education about research or using research findings in their basic nursing education, nor have they had continuing education on EBP in the workplace [5]. Education and training were described as necessary to facilitate EBP in our study; however, research has shown that implementation is complex and education alone will not ensure research findings are used in practice [61, 62], particularly where respect for social order is a strong influence on how people act.

Organizational characteristics such as location, size, infrastructure and resources, have been associated with knowledge and attitudes towards EBP in China [5]. Healthcare inequities between more-developed urban provinces and less-developed rural provinces contribute to differences in organizational characteristics [11, 12]. Consistent with our study, survey research has revealed nurses in rural hospitals are less knowledgeable about EBP than nurses in tertiary hospitals [5, 36].

Evidence based practice is a complex process that requires multidimensional change at the individual, organizational and cultural levels. This study brings us closer to understanding some of the changes that could support nurses to use rigorous research evidence to guide their clinical practice in China. With the ubiquitous use of electronic internet devices, nurses require critical appraisal skills to safely and judiciously use evidence that is readily available on the internet. Evidence-based guidelines in Chinese are needed to provide nurses access to research knowledge for making practice decisions. Further research is required into the process of translating and adapting clinical practice guidelines and other knowledge translation tools into Chinese language and context to support nurses use evidence based knowledge in practice.

Our study acknowledged the importance of nursing leadership in creating a work environment that supports EBP. However it also demonstrates the complexity of the leadership process to influence change. Further research is required to understand leadership in China and how to effectively develop nursing leaders to facilitate and support the implementation of evidence based practices.

### Study strengths and limitations

We used a small sample in order to perform in-depth qualitative interviews with bilingual translation during the interviews, allowing for rich data collection and interpretations of meanings. Nurses from multiple levels of the organizations were interviewed providing insights into the structures and processes that impact direct patient care. While translation and back-translation processes were conducted, other semantic meanings and cultural norms may have been present and not captured.

## Conclusions

Our findings from Hunan province provide an important understanding of the barriers and facilitators of EBP in nursing from the narratives of three stakeholder groups: staff nurses, head nurses and nursing directors. While several parallels to previous research were found, this study adds to the knowledge base about factors related to EBP in different geographic and contextual settings. To realize a successful implementation of EBP in mainland China, it is important that strategies consider nurses’ perspectives from multiple levels within an organization. Findings are important for international comparisons so that strategies can be developed for nurses to deliver evidence-based practice and improve the quality of patient care.

## Additional file

Additional file 1: Interview Schedules. Semi-structured interview guide in English and Chinese. (DOCX 47 kb)

## Abbreviations

Apps: Social media applications
EBP: Evidence-based practices
