# Supplementary material for: When east meets west: a qualitative study of barriers and facilitators to evidence-based practice in Hunan China
Source: BMC Nurs. 2018 Jun 20;17:26. doi: 10.1186/s12912-018-0295-x (PMC6011581; doi:10.1186/s12912-018-0295-x)
Supplement: Supplementary file 1 — Interview Schedules. Semi-structured interview guide in English and Chinese. (DOCX 47 kb) [file 12912_2018_295_MOESM1_ESM.docx]

**Additional file 1**

**China Study Interview Schedules**

**Introduction/preparation for interview**

**访谈简介／准备**

**3 respondent groups:**

1. Leaders and managers of nurses/nursing at a clinical practice level
2. Facilitators responsible for supporting evidence-based practice
3. Executive Nurses/Directors of Nursing/Directors of Patient Care or Directors of Community Care (with responsibility for nursing)

3个受访组

a）临床实践层面的领导者和管理者

b）循证实践的促进者

c）执行护士/护理部主任/科护士长或社区护理部主任

1. Identify interview schedule appropriate to respondent group i,ii or iii above.
2. Interviews to commence with a summary of the project and confirm confidentiality of responses received.
3. All respondents are to be asked for permission to record interview with digital recorder. [If refused, notes are to be taken throughout.]
4. Interviewer obtains verbal consent / written consent from respondent that confirms understanding and participation of above.
5. All interviews to be digitally recorded for verbatim transcription (if agreed).
6. All interviews to be conducted on a 1-1 basis (one interviewer and one respondent).
7. Interview schedule should take approximately 40-50 minutes to complete
8. 对上述3个受访组确定合适的访谈计划。
9. 访谈从项目的概要开始，并承诺对所收集的信息保密。
10. 获得所有受访者的允许后方可录音。[如拒绝，全程做好记录]。
11. 访谈者获得受访者的口头同意或书面同意，并确认其理解和参与。
12. 所有的访谈内容将逐字转录成数字信息（在受访者同意的基础上）。
13. 所有的访谈是一对一（一个访谈者和一个受访者）。
14. 访谈约需40-50分钟。

**Interview Schedule (a): Leaders and managers of nurses/nursing at a clinical practice level**

访谈计划（a）：临床实践层面的领导者和管理者

1. Clarification of role and position in the organization

Can you briefly tell us a bit about your own role, what you are responsible for and who you are accountable to within the organization?

明确组织中的角色和地位

你能简要地告诉我们一些关于你承担的角色、职责、在组织中对谁负责？

1. Knowledge and decision making

知识与决策

As we have explained, we are interested in the roles that managers, leaders and facilitators play in supporting the implementation of evidence-based practice in nursing. We would like to start by asking you some general questions about decision-making in your ward/unit and what knowledge is used to inform this.

正如前面所介绍，我们对管理者、领导者和促进者在支持循证实践实施中所发挥的作用很感兴趣。首先问有关于你病房/单元在决策中的一些常见问题，以及你运用哪些知识来形成决策？

- Thinking about day-to-day decision making on the ward/unit, what are the main sources of knowledge that are used? [Prompts: clinical standards and guidelines; advice from colleagues; accessing networks; clinical judgment, policies and protocols etc.]

想一想病房里一天天的决策，所用到的知识的主要来源是什么？ [提示：临床标准和指南；同事的建议；网络资源；临床判断、政策和方案等。]

- How do you know that these are up to date and trustworthy?

你怎么知道这些知识新且可信？

- And how do you assess whether appropriate clinical decisions are being made? [Prompts: what conversations might they have with nursing staff, what questions would they ask, what would they look for in the nursing notes?]

你如何评估临床决策是否恰当？[提示：他们可能会跟护理人员说些什么，他们会问些什么问题，他们会在护理记录中寻找什么？]

1. Experiences of evidence-based practice

循证实践经验

Thinking more specifically about evidence-based practice and strategies for implementing evidence into practice:

请认真思考：循证实践和从证据到实践的实施策略

- How well do you think this currently happens in your ward/unit?

你认为循证实践在你病房/单元里实施的情况怎么样？

- Can you describe an example of EBP from your ward/unit?

能举出你病房/单元循证实践的例子吗？

- - Explore sources of evidence used and how they were accessed and appraised

探讨所使用证据的来源，以及如何获取及评价它们？

- - Discuss processes of implementation; who was involved; roles and relationships; what worked well/less well

讨论实施的过程，参与者及其角色，参与者之间的关系；哪些做得好/欠佳？

- - What types of methods were used e.g. PDSA, audit and feedback, educational events, reminders?

采用什么方法？如PDSA，旁听和反馈，教育活动，提示？

- - What outcomes were achieved; how was evaluation undertaken and by whom?

取得了什么成果，如何进行评估，由谁来评估？

- - Were specific models and frameworks used to guide implementation and evaluation e.g. K2A, PARIHS, CFIR, others?

是否采用特定的模型和理论框架（如K2A，健康服务领域研究成果应用的行动促进(promoting action on research implementation in health service, PARIHS)，CFIR或其他的）来指导实施与评价？

- What approach do you take with staff to ensure the application of evidence in their practice? [Prompts: relationships with staff; measuring outcomes]

你和工作人员采取什么的方法来确保证据在实践中的运用？[提示：与工作人员的关系；测量结果]

- From your experience, what would you say are some of the key factors that:
  - Enable the implementation of EBP?
  - Present barriers or challenges in implementing EBP?

根据你的经验，

你认为哪些关键因素使得循证实践得以实施？

在循证实践实施中遇到的障碍或挑战是什么？它们的影响因素是什么？

- Do you think EBP is an organizational priority?

你认为循证实践是一个组织之首选吗？

- - If yes, what structures and processes are in place to support EBP? [Prompts: are there systems to enable access to evidence; educational programmes for staff; facilitator support; audit and feedback systems etc.]

如果是，用什么样的结构和程序来支持循证实践？[提示：是否有能获得证据的体系；工作人员的教育项目；促进者支持；旁听和反馈体系等]。

- - If no, explore reasons why they think this.

如果不是，探讨他们为何这样认为（为什么）。

1. Own role in evidence-based practice

在循证实践中，你自己的角色

- How do you see your own role in supporting the use of evidence in nursing practice?

怎么看待你自己在护理实践中支持使用证据的角色？

- - Can you describe specific activities you undertake to support EBP?

你能描述一下你支持循证实践的具体活动？

- - How do you encourage staff to have an enquiring approach to their clinical practice?

[Prompts: are there distinct leadership and facilitation practices within their role?]

你是如何鼓励工作人员使用咨询方法到实践中去？[提示：是否有优秀的领导力和促进措施？]

- Have you had any education or training in EBP?
  - Within the organization or as part of an external course.

你接受过循证实践教育或培训吗？

正规培训或者组织外的部分培训 额外课程的一部分，

- Who are the key people that you engage with in relation to leading and supporting evidence-based practice?
- 在你从事领导和支持循证实践有关的工作中，谁是关键性人物？
  - Do you see a distinction between your own role and others who may be involved in supporting EBP, for example, dedicated practice facilitators or educators?
  - 你是否看到你自己和其他人参与支持循证实践的区别，如潜心于循证实践促进者或教育者。
- Would you say that you practice evidence-based decision making as a clinical manager/leader? [Prompt: do they use evidence to inform their management or leadership practice?]

你认为你是作为一个临床管理者或领导者来开展循证决策的吗？[提示：他们是否使用证据来指导他们的管理或领导？]

1. Additional thoughts and comments

其他想法和建议

- Finally, if there was one thing that you could change to improve EBP, what would that be?
- Do you have any additional thoughts or comments you would like to make in relation to EBP in nursing and the roles that different people play in supporting this?

最后，如果有一件事情，你可以改变它来提高循证实践，是件什么事情 ？

- 有关于循证实践，以及不同人在支持循证实践中发挥的作用，你是否有其他想法或建议？

**Interview Schedule (b): Facilitators responsible for supporting evidence-based practice**

访谈计划（b）：循证实践促进者

1. Clarification of role and position in the organization

明确在组织中的作用和地位

Can you briefly tell us a bit about your own role, what you are responsible for and who you are accountable to within the organization?

你能简要地告诉我们关于你的角色、你负责什么，在组织中你对哪些人负责？

1. Knowledge and decision-making

知识与决策

As we have explained, we are interested in the roles that managers, leaders and facilitators play in supporting the implementation of evidence-based practice in nursing. We would like to start by asking you some general questions about how the nursing staff you work with make decisions and what knowledge is used to inform this.

正如前面所介绍，我们对管理者、领导者和促进者在循证支持实践方面发挥的作用很感兴趣。我们首先将问你一些常见的问题，这些问题是关于与你一起工作的护士是如何决策以及哪些知识被用于形成决策的？

Thinking about day-to-day decision making at a clinical level, what are the main sources of knowledge that are used? [Prompts: clinical standards and guidelines; advice from colleagues; clinical judgement, policies and protocols etc.]

想一想病房里一天天的决策，所用到的知识的主要来源是什么？[提示：临床标准和指南；同事的建议；临床判断、临床政策和临床方案，等]

- What is your sense of the knowledge sources that are used [Prompts: are they reliable, up to date, trustworthy?]

你对你常使用的知识来源的感觉是什么？[提示：他们是否可信，是否是最新的，或是否值得信赖？]

- And how do you assess whether appropriate clinical decisions are being made? [Prompts: what conversations might they have with nursing staff, what questions would they ask, what would they look for in the nursing notes?]

你如何评估临床决策是否恰当？[提示：他们与护士可能会说什么？他们会问什么问题？他们会在护理记录中查找什么？]

1. Experiences of evidence-based practice

循证实践经验

Thinking more specifically about evidence-based practice and strategies for implementing evidence into practice:

请认真思考：循证实践和从证据到实践的实施策略：

- How well do you think this currently happens in this organization?

你认为循证实践在你病房/单元里实施的情况怎么样（有多好）？

Can you describe an example of how you have facilitated EBP?

你能举一个例子说明你是如何促进循证实践的？

Explore sources of evidence used and how they were accessed and appraised

在探讨已使用证据的来源时，你们是如何评估和评价它们？

- How well do you think this currently happens in your ward/unit?

你认为循证实践在你病房/单元里实施的情况怎么样？

- Can you describe an example of EBP from your ward/unit?

能举出你病房/单元循证实践的例子吗？

Discuss processes of implementation; who was involved; roles and relationships; what worked well/less well

讨论实施的过程，哪些人参与，他们的职责和关系；哪些做得好，哪些做得欠佳？

What types of methods were used e.g. PDSA, audit and feedback systems , educational events, reminders?

应用什么方法？例如PDSA，旁听和反馈，教育活动，提醒？

- - What outcomes were achieved; how was evaluation undertaken and by whom?

取得了什么成果，如何评估？哪些人评估？

- - Were specific models and frameworks were used to guide implementation and evaluation e.g. K2A, PARIHS, CFIR, others?

是否有具体的模型和框架（如K2A，PARIHS，CFIR，和其他的？）用于指导实施和评估？

- From your experience, what would you say are some of the key factors that:
  - Enable the implementation of EBP?
  - Present barriers or challenges in implementing EBP?

根据你的经验，

你认为哪些关键因素使得循证实践得以实施？

在循证实践实施中遇到的障碍或挑战是什么？他们的影响因素是什么？

- Do you think EBP is an organizational priority?

你认为循证实践是一个组织之首选吗？

- - If yes, what structures and processes are in place to support EBP? [Prompts: are there systems to enable access to evidence; educational programmes for staff; facilitator support; audit and feedback systems etc.]

如果是，用什么样的结构和过程来支持循证实践？[提示：是否有能获得证据的体系；工作人员的教育项目；促进者支持；旁听和反馈体系等。]

- - If no, explore reasons why they think this.
  - 如果不是，探讨他们为何这样认为（为什么）。
- What approach do you take with staff to ensure the application of evidence in their practice? [Prompts: relationships with staff; measuring outcomes]

你和工作人员采取什么的方法来确保证据在实践中的运用？[提示：与工作人员的关系；测量结果]

1. Own role in evidence-based practice

在循证实践中，你自己的角色

- How do you see your own role in supporting the use of evidence in nursing practice?

怎么看待你自己在护理实践中支持使用证据的角色？

- - Initiating projects or responding to requests for help?

启动项目或提供帮助？

- - Key activities undertaken as part of the role?

作为角色的一部分，进行了哪些关键活动？

- - How do you encourage staff to have an enquiring approach to their clinical practice?

你是如何鼓励工作人员掌握临床实践证据的查询方法的？

- What preparation have you had for the role of facilitator?
  - Formal training in facilitation and/or EBP?
  - Supervision and mentorship in the role?

作为循证实践促进者，你为该角色做了哪些准备？有无循证实践的正式培训，有无监督者和指导者？

- Are you working as a sole facilitator or as part of team?

你是否作为唯一的循证实践的促进者，或团队成员之一来工作？

- Who are the key people that you engage with in relation to facilitating EBP?

谁是使你从事促进循证实践相关工作关键人？

- And thinking about EBP at a clinical level, how does your role relate to that of the clinical leader/manager?

在临床上思考循证实践，你的角色是如何与临床领导/管理者的角色进行协调的？

- Would you say that you practice evidence-based decision making as a facilitator? [Prompt: do they use evidence to inform their facilitation practice?]

你认为你是作为一名促进者来实施循证决策的吗？[提示：他们是否使用证据来指导他们促进实践？]

1. Additional thoughts and comments

其他想法和建议

- Finally, if there was one thing that you could change to improve EBP, what would that be?
- Do you have any additional thoughts or comments you would like to make in relation to EBP in nursing and the roles that different people play in supporting this?

最后，如果有一件事情，你可以改变它来提高循证实践，是件什么事情 ？

在护理中有关于循证实践，以及不同人在支持循证实践中发挥的作用，你是否有其他想法或建议？

**Interview Schedule (c): Executive Nurses/Directors of Nursing**

访谈计划：执行护士/护士长(护理部主任）

1. Clarification of role and position in the organization

明确在组织中的作用和地位

Can you briefly tell us a bit about your own role, what you are responsible for and who you are accountable to within the organization?

你能简要地告诉我们关于你的角色、你负责什么，在组织中你对哪些人负责？

1. Knowledge and decision-making

知识和决策

As we have explained, we are interested in the roles that managers, leaders and facilitators play in supporting the implementation of evidence-based practice in nursing. We would like to start by asking you some general questions about how nursing staff in the organization make decisions and what knowledge is used to inform this.

正如我们前面解释过的，我们对管理者、领导者和促进者在循证支持护理实践方面发挥的作用很感兴趣。我们首先将问你一些常见的问题，这些问题是关于与你一起工作的护士是如何决策以及哪些知识被用于形成决策？

- Thinking about day-to-day decision making at a ward/unit level, what are the main sources of knowledge that are used? [Prompts: clinical standards and guidelines; advice from colleagues; clinical judgement, policies and protocols etc.]

想一想病房里一天天的决策，所用到的知识的主要来源是什么？[提示：临床标准和指南；同事的建议；临床判断、临床政策和临床方案等]

- How do you know that these are up to date and trustworthy?

你怎么知道这些知识是最新且可信？

- And how do you assess whether appropriate clinical decisions are being made? [Prompts: what conversations might they have with nursing staff/managers, what questions would they ask, what would they look for in the nursing notes?]

你如何评估临床决策是否恰当？[提示：他们可能会和护士/护理管理者说什么，他们会问些什么问题，他们会在护理记录中寻找什么？]

1. Experiences of evidence-based practice

循证实践经验

Thinking more specifically about evidence-based practice and strategies for implementing evidence into clinical practice:

请认真思考：循证实践和从证据到实践的实施策略：

- How well do you think this currently happens in this organization?

你认为循证实践在你病房/单元里实施的情况怎么样（有多好）？

- How well do you think this currently happens in your ward/unit?
  - Within nursing specifically?

你认为在护理专业领域，循证实践的现状如何（有多好）？

- - And more generally within other disciplines and at an inter-disciplinary level?

你认为在其他学科和交叉学科水平，循证实践现状如何（有多好）？是否更普遍？

- Is EBP an organizational priority?

你认为循证实践是一个组织之首选吗？

- - If yes, what structures and processes are in place to support EBP? [Prompts: are there systems to enable access to evidence; educational programmes for staff; facilitator support; audit and feedback systems etc.]

如果是，用什么样的结构和过程来支持循证实践？[提示：是否有能获得证据的体系；工作人员的教育项目；促进者支持；旁听和反馈体系等。]

- - If no, explore reasons why they think this.
  - 如果不是，找出他们这样想的原因（为什么）
- How do you monitor and evaluate the extent to which EBP is occurring at a clinical and organizational level?

你是如何监督和评估在临床和组织层面实施的临床实践程度的？

- What approach do you take with staff to ensure the application of evidence in their practice? [Prompts: relationships with staff; measuring outcomes]

你和工作人员采取什么的方法来确保证据在实践中的运用？[提示：与工作人员的关系；测量结果]

- Who are the key people involved in leading and supporting EBP?

谁是使你领导并支持临床护理实践工作的关键人？

- From your experience, what would you say are some of the key factors that:
  - Enable the implementation of EBP?
  - Present barriers or challenges in implementing EBP?

根据你的经验，

你认为哪些关键因素使得循证实践得以实施？

在循证实践实施中遇到的障碍或挑战是什么？它们的影响因素是什么？

1. Own role in evidence-based practice

在循证实践中，你自己的角色

- How do you see your own role in supporting the use of evidence in nursing practice?

怎么看待你自己在护理实践中支持使用证据的角色？

- - Can you describe specific activities you undertake to lead and support EBP?

你能描述一下你领导和支持循证实践的具体活动吗？

- - How do you encourage staff to have an enquiring approach to their practice?

[Prompts: are there distinct leadership and facilitation practices within their role?]

你如何鼓励工作人员掌握查询临床实践证据的？[提示：他们在领导和促进实践中的作用是否明显？]

- Who are the key people that you engage with in relation to leading and supporting evidence-based practice?

谁是使你从事领导和支持循证实践有关的工作的关键人？

- - Do you see a distinction between your own role and others who may be involved in supporting EBP, for example, dedicated practice facilitators or educators?

o你是否看到你自己和其他人参与支持循证实践的区别，如潜心于循证实践促进者或教育者。

- Would you say that you practice evidence-based decision making as an executive nurse? [Prompt: do they use evidence to inform their management or leadership practice?]

你认为你是作为一名执行护士来开展循证决策的吗？[提示：他们是否使用证据来指导他们的管理或领导？]

1. Additional thoughts and comments

其他想法和建议

- Finally, if there was one thing that you could change to improve EBP, what would that be?
- Do you have any additional thoughts or comments you would like to make in relation to EBP in nursing and the roles that different people play in supporting this?

最后，如果有一件事情，你可以改变它来提高循证实践，是件什么事情 ？

在护理中有关于循证实践，以及不同人在支持循证实践中发挥的作用，你是否有其他想法或建议？
